# Unilateral triplicate ureter with ipsilateral ureterocele a case report

**DOI:** 10.1016/j.ijscr.2020.04.048

**Published:** 2020-05-11

**Authors:** Mohammad Al-Zubi, Anas Al Faqieh, Oroub Altamimi, Soha Albeitawi

**Affiliations:** a Department of Urology, Faculty of Medicine, Yarmouk University, Irbid, Jordan; b Faculty of Medicine, Yarmouk University, Irbid, Jordan; c Department of Gynacology, Yarmouk University, Irbid, Jordan

**Keywords:** Triplicate ureter, Ureterocele, Smith classification, Congenital abnormality

## Abstract

**Introduction:**

Triplicate ureter is a rare congenital anomaly of the upper urinary tract.

**Presentation of case:**

In this case we present a triplicate ureter with ipsilateral ureterocele in a 9 years old boy. After literature review we found out this presentation very rare.

**Discussion:**

Ureteral triplication classified by Smith into 4 subtypes, and usually associated with other congenital abnormality, most common one is contralateral ureteric duplication. Usually this anomaly is asymptomatic.

We present a type 3 ureteral triplication.

**Conclusion:**

Although triplicate ureter is a rare congenital anomaly, we must keep it in our differential diagnosis of unexplained urinary symptoms.

## Highlights

• Triplicate ureter is a very rare congenital abnormality of upper urinary tract.
• Usually asymptomatic.
• Associated with other congenital abnormality.

## 1 Introduction

Triplication of the ureter is a very rare upper urinary tract abnormality, there were nearly 100 cases that have been reported from 1870 to 2004 [1]. Smith classification is used to classify ureter triplication to the following subtypes [2], (1) Complete triplication: where three ureters from the kidney drain separately into the bladder or ectopically. This is the most common type, making up 35% of all triplications. (2) Double ureter with one bifid: where there are three ureters from the kidney and two join draining into two ureteric orifices in the bladder. This accounts for 21% of triplications. (3) Trifid ureters (as in our case): where the three ureters join into a single orifice. 31% of triplications are type 3. (4) Double ureters from the kidney, with one bifurcating as an inverted Y draining into three orifices in the bladder. We report a case of type 3 ureteral triplication in association with ipsilateral ureterocele. This is an interesting case as after review of literature we found out this presentation is unusual. This work has been reported in line with the SCARE criteria [3].

## 2 Case presentation

A 9-year-old boy was admitted complaining of dysuria associated with sudden, intermittent, left flank pain, with no history of previous similar attacks. Physical examination showed stable vital signs, body weight of 29 kg, and no abdominal masses.

Urine analysis and kidney function test were unremarkable.

**Fig. 1:**
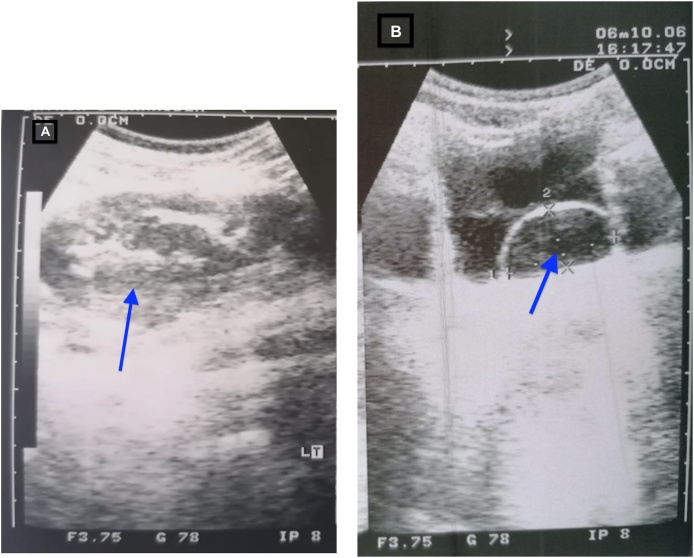
A: Urinary tract ultrasound showing left kidney hydronephrosis (blue arrow) B: urinary tract ultrasound showing left sided large urinary bladder ureterocele (blue arrow).

**Fig. 2:**
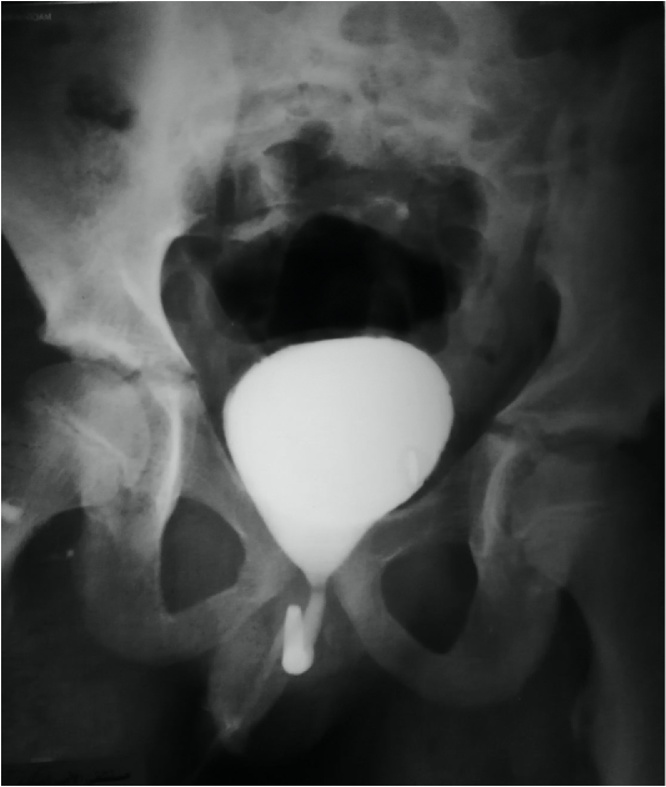
MCUG (micturating cystourethrogram) showing normal bladder shape with no reflux bilaterally.

**Fig. 3:**
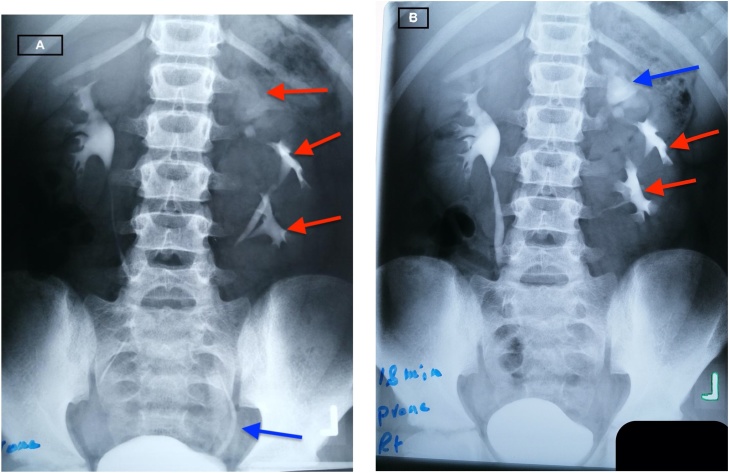
A: IVU (intravenous urogram) showing normal right kidney with 3 moieties in the left kidney (red arrows) draining into urinary bladder as one ureter (blue arrow). B: Intravenous urogram showing dilated upper moiety (blue arrow) compared to the mid and lower moieties (red arrows) on left kidney.

Radiological investigations started with Ultrasonography for the urinary tract (Fig. 1) which showed hydronephrosis of the left kidney with cystic lesion in the bladder mostly represents a ureterocele, then MCUG (Micturating cystourethrogram) (Fig. 2) was done to rule out vesicoureteral reflux, which showed normal bladder shape with no reflux on both sides. Based on the above results, MAG-3 (Mercaptuacetyltriglycine) was done to rule out pelvic-ureteric junction obstruction as a cause of hydronephrosis, it showed that both kidneys were normally located and had symmetrical size, with split function in the right 52% and in the left 48%, the left kidney is likely composed of 3 moieties: the upper moiety is so small with poor uptake and excretion, the left middle and lower moieties represented most of the left kidney and showed good, homogeneous uptake and excretion with smooth contour, without evidences of further segmentation. After that IVU (intravenous urogram) (Fig. 3) was performed and showed a normal collecting system on the right side and a type 3 ureteral triplication with an abnormal triplication of the left kidney with dilated upper moiety. The three ureters draining from the left side kidney end into a single ureteric orifice inside urinary bladder, with the lower 2 joining at the level of L5.

Due to the above investigations, cystoscopy was done and showed normal urinary bladder apart from large ureterocele, which was large enough to be managed endoscopically. Then decision was made to perform left partial nephroureterectomy for the upper moiety (to avoid pain from recurrent infection as upper moiety was small with poor uptake and excretion on MAG3) and to excise the ureterocele. After the operation, the patient did well and was discharged home after 15 days without any complications. Histopathology came back as left ureter excision with chronic inflammation and left partial nephrectomy with acute and chronic pyelonephritis changes.

**Fig. 4:**
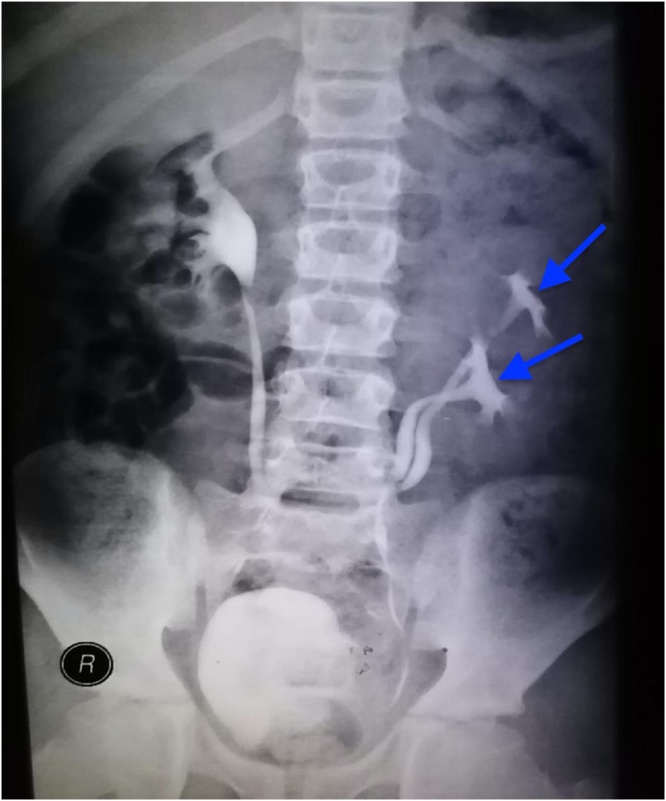
Post operative follow up intravenous urogram showing normal excretory function for the upper and lower moieties (blue arrows) in the left kidney.

A Follow-up IVU one-month post operation showed normal nephrogram bilaterally with incomplete duplication of left kidney without obstruction (Fig. 4).

Written and oral consent for use of the data and photos for research project and educational purposes was obtained from the parents.

## 3 Discussion

Ureteral triplication is a rare congenital anomaly of upper urinary tract [4]. Ureteral triplication is more common in females and is commonly located at the left side, as in our case [5].

Triple ureters may be responsible for recurrent urinary tract infections, incontinence, enuresis or pain caused by ureterocele but most of the time this anomaly is asymptomatic and detectedincidentally during the investigation of other conditions, and this can explain its late presentation [4]. in childhood UTI, the use of renal ultrasound and a focused ‘top down’ investigation accordingly (MCUG, MAG3, DMSA (dimercaptosuccinic acid) and other investigations) approach is likely to identify the vast majority of children who require intervention [6]. Ureteral triplication comes with other congenital abnormality; the most common abnormality is ureteral duplication in the contralateral ureter (37%), ureteral ectopia (28%) and renal dysplasia (8%) [5]. Occasionally ureterocele, vesicoureteric reflux, and angiomas, are other associations [7]. Ureteric duplication and triplication have been explained by multiple ureteric buds arising independently from the Wolffian duct, and/or early fusion of one or more ureteral buds [8].

Most cases of ureteral duplication conform to the Weigert-Meyer law [9], which states that the ureter from the upper pole of the kidney is incorporated into the bladder more caudally and medially than the lower pole ureter. However, the Weigert-Meyer principle does not apply to all patients with ureteral triplication [10]. Urinary tract ultrasound and computed tomography are useful in the diagnosis of triplicate ureters, but intravenous urography may be more useful in completely defining the anatomy [4].

## 4 Conclusion

Ureteral triplication is a very rare congenital anomaly, and because of the paucity of presenting symptoms, this can delay its diagnosis. Our recommendation is to keep this anomaly in the differential diagnosis of recurrent UTI, incontinence and recurrent urolithiasis.

## Declaration of Competing Interest

No conflicts of interest.

## Sources of funding

No sources of funding for my research.

## Ethical approval

This research did not need ethical approval.

## Consent

Fully informed written consent was signed from the parents of the patient.

## Author contribution

**MOHAMMAD. AL-ZUBI**: study design, data collection, data analysis and writing the paper.

**ANAS AL FAQIEH**: data collection, and writing the paper.

**OROUB ALTAMIMI**: data analysis and writing the paper.

**SOHA ALBEITAWI**: study design, data collection.

## Registration of research studies

NA.

## Guarantor

MOHAMMAD. AL-ZUBI.

## Provenance and peer review

Not commissioned, externally peer-reviewed.
